# Superhydrophobicity Effects on Spheroid Formation and Polarization of Macrophages

**DOI:** 10.3390/ph17081042

**Published:** 2024-08-07

**Authors:** María del Carmen Morán, Francesca Cirisano, Michele Ferrari

**Affiliations:** 1 Departament de Bioquímica i Fisiologia, Secció de Fisiologia—Facultat de Farmàcia i Ciències de l’Alimentació, Universitat de Barcelona, Avda. Joan XXIII, 27-31, 08028 Barcelona, Spain; 2 Institut de Nanociència i Nanotecnologia—IN2UB, Universitat de Barcelona, Avda. Diagonal, 645, 08028 Barcelona, Spain; 3 CNR-ICMATE Istituto di Chimica della Materia Condensata e di Tecnologie per l’Energia, Via De Marini, 6, 16149 Genova, Italy; francesca.cirisano@cnr.it

**Keywords:** activation, cell morphology, macrophages, polarization, spheroids, superhydrophobicity, surfaces

## Abstract

The interaction of biomaterials with the immune system is ruled by the action of macrophages. The surface features of these biomaterials, like wettability, which is an expression of chemical composition, texture, and geometry, can affect macrophages response. Such surface parameters can be then efficiently exploited to improve biocompatibility by lowering undesired immunological reactions and at the same time creating the substrate for positive interactions. In this work, the preparation and physicochemical characterization of highly water-repellent surfaces to develop and characterize 3D spheroids derived from monocyte-macrophages (RAW 264.7 cell line) has been carried out. As a measure of cell viability over time, the obtained aggregates have been transferred under standard 2D cell culture conditions. Significant changes on the morphology-associated polarization of the derived cellular entities have been evaluated at the nanoscale through 3D profilometry. The results suggested that the spheroid formation using highly repellent substrates induced the activation of M2-type cells. This simple and cost-effective approach can be used for preparing M2-based macrophages for regenerative purposes.

## 1. Introduction

The interaction between cells and surfaces is a critical area of study in biomedical research, influencing various applications from tissue engineering to implant design [1]. Cells respond to the physical and chemical properties of the surfaces they encounter, which can dictate their behavior, growth, and function [2]. Even key phenomena occurring in biology can be related to interfacial processes involving cell/tissue and surfaces considerable as the basis for the design of many artificial biomaterials and their applications [3,4,5,6].

A biocompatible material can be defined as capable of providing suitable support for cell activity, also stimulating molecular and mechanical phenomena. For improvement of tissue regeneration, avoiding side effects without producing negative responses in the host at the local or systemic level is required [7,8,9]. The biocompatibility assessment of biomaterials has been demonstrated to be highly dependent on their surface properties like stiffness, surface charge, chemical functionalities, roughness, and wettability, beyond cell type and culture conditions as key parameters for their modulation [10].

Macrophages, a type of immune cell, play a significant role in the body’s response to foreign surfaces. When materials are introduced into the body, macrophages are among the first cells to interact with them. These interactions can lead to inflammation and foreign body reactions, as well as ultimately determining the success or failure of implanted materials [11]. The high plasticity of macrophages makes their phenotypes and functionalities sensitive to different environmental changes [12]. When activated, two groups of macrophages can be identified as a function of their different biological activity: M1, the “classically activated” pro-inflammatory macrophages, and M2, the “alternatively activated” anti-inflammatory macrophages [13].

Under well-defined in vitro conditions, a single-cell approach can be regarded as a simple model to suitably predict the complex foreign body reaction. To this aim, human-monocyte-derived macrophages, human monocyte cell lines, mouse bone-marrow-derived macrophages, or murine macrophage cell lines are used as culture models. The literature contains ample evidence of the induction of in vitro polarity to M1 or M2 phenotype by supplementation of the culture medium with lipopolysaccharide and interferon gamma or with interleukin-4 and interleukin-13, respectively [14,15]. However, in addition to classical biochemical responses, macrophages also respond to mechanical stimuli such as surface chemistry, pore size, strain, and topography [16,17,18]. Macrophages have been demonstrated to exhibit stronger inflammatory responses in vitro depending on the 2D substrate stiffness [19,20,21,22,23], while a 3D matrix culture environment can address cells to the opposite result. Matrix stiffness has been found to orient in vitro and in vivo toward a more immunosuppressive, M2-like phenotype [24,25,26].

Surface properties are regarded with growing interest since their features meet the requirements in view of more reliable in vitro tests based on 3D aggregates, an innovative approach in comparison with traditional ones [27,28]. Spheroids, which are three-dimensional aggregates of cells, provide a more physiologically relevant model for studying cell behavior compared to traditional two-dimensional cultures. Recent studies have demonstrated how superhydrophobicity can be used to prepare 3D spheroids [29,30,31]. Interestingly, the migrated cells demonstrated increased cell attachment and proliferation in comparison with those growth under conventional 2D culture [31]. Thus, superhydrophobicity can be effectively used as a platform for 3D spheroid formation and recovery, as well as for promoting added value to the biological characteristics in its application for regenerative purposes.

In view of potential applications on cell-based immunotherapy, the objective of this study was to verify the effect of growth in 3D spheroids generated on highly hydrorepellent surfaces on the morphology, viability, and polarization of derived cell entities. In this work, the preparation and physicochemical characterization of highly water-repellent surfaces to develop and characterize 3D spheroids derived from monocyte-macrophages (RAW 264.7 cell line) was carried out. The effectiveness of the obtained spheroids in being in recovery and transferred to make possible the growth of the derived cell entities under 2D monolayer culture as a measure of cell viability was performed. Changes on the morphology-associated polarization of the derived cellular entities after spheroid formation and recovery was evaluated at the nanoscale by means of 3D profilometry.

## 2. Results

### 2.1. Surface Characterization of Superhydophobic Coating

**Figure 1:**
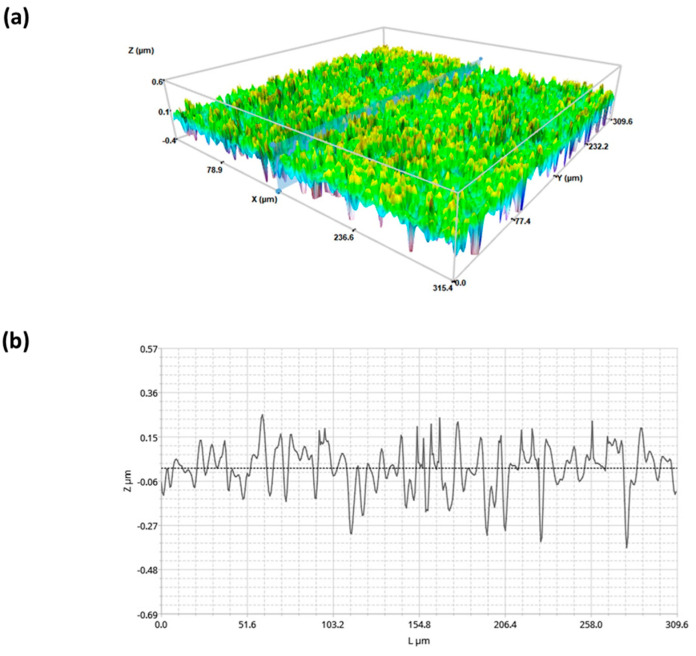
Three-dimensional detail of the superhydrophobic surface (**a**) and its roughness acquired by interferometric and confocal profilometry at 20× (**b**). Average roughness, Sa, 170 nm.

The superhydrophobic coatings (SH) were deposited by a spray technique using a fluorinated polymers/silica nanoparticle dispersion and were studied by focusing on their wettability, surface geometry, and roughness (Sa). The coated surfaces showed contact angles (CA) higher than 160° with low hysteresis (<5°), resulting in drops rolling off the surface. From the topography studies, carried out by 3D confocal and interferometric profilometry, there appeared a double-scale nanometric roughness with an average value of 170 nm, with homogeneously distributed superhydrophobic properties. A representative 3D image at 20× and the related profile are shown in Figure 1.

The performance of SH surfaces is highly dependent on both the concentration of silica nanoparticles and the type of fluorinated polymer used. In fact, surface roughness and then hydrophobicity can be modulated by acting on concentration. From low concentration, an SH effect is unable be present. However, when it is too high, the aggregate formation could have a negative effect on the homogeneity, the overall reproducibility, and mechanical resistance. Optimal performance requires balancing these variables to achieve the desired level of hydrophobicity; mechanical durability; chemical resistance; and, as in our case, transparency. Tailoring the SH surface composition to specific application requirements is essential for maximizing its effectiveness and longevity.

### 2.2. Cell Behavior in Different Substrates 

**Figure 2:**
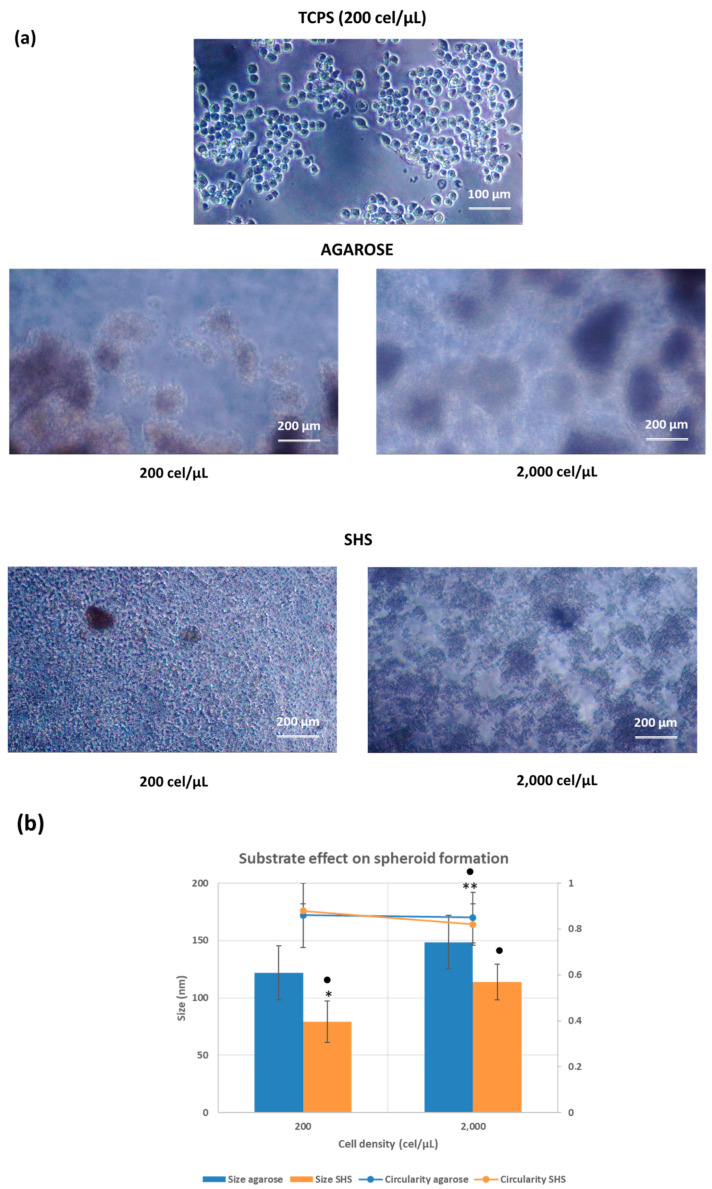
Phase contrast images on RAW264.7 cells cultured in TCPS, agarose, and highly water-repellent surfaces (SHS) after 48 h of incubation based on initial cell density (200 or 2000 cel/μL) (**a**). Circularity and size distribution (**b**) based on initial cell density (200 or 2000 cel/μL) on agarose and SHS. The results are reported as the average of more than 10 individual spheroids ± standard deviation. * *p* < 0.05 and ** *p* < 0.001 indicate significant differences between values as a function of the initial cell density for the same substrate. • *p* < 0.05 indicates significant differences between values as a function of the substrate for the same initial cell density.

Cell culture in tissue culture polystyrene (TCPS) plates was used to assess the morphological characteristics of these macrophage-type cells in controlled 2D features. RAW 264.7 cells have both loosely adherent cuboidal or spindle-shaped cells and rounded viable cells (Figure 2a). The typical cell morphology appeared to be circular with a smooth shape. The capability of RAW 264.7 cells to form spheroid-like structures was assessed by seeding them at two different cell densities (200 and 2000 cell/μL) on the top of agarose as a base layer. The lower density corresponded to the cell density used under standard 2D conditions, for comparative purposes. The highest cell density was used to promote the 3D formation, as has been done in previous studies [30,31]. Because of their biophysical properties that mimic the extracellular matrix (ECM), hydrogels are recognized as suitable platforms for promoting spheroid formation [32,33]. Agarose, among all the compounds that can form a hydrogel, is natural, inexpensive, non-toxic, and effective in repelling cells, resulting in spheroid formation. Moreover, agarose hydrogels have good permeability in terms of nutrients and drugs, which is crucial in the study of spheroid applications [34,35].

When using the agarose platform, the 3D spheroids are usually formed by the cells forming aggregates or a cluster of cells resembling grapes as a result of gravitational pull, with the agarose meniscus forming a focus point [36]. The results shown in Figure 2a demonstrate that RAW 264.7 cells showed diffuse structures of big dimensions and aggregates of different compactness and sphericity at the two assayed concentrations. The effectiveness of the agarose-induced 3D spheroids was highly dependent on the cell line type, as has been recently reported in previous results [30]. These results are in light of previous results in the literature [37,38] where, despite the non-adherent properties of agarose (contact angle (CA) = 90° and contact angle hysteresis (CAH) > 10°), the derived hydrogels have demonstrated limitations on the growth of tumoral cells by lacking the activation of specific tumoral signaling pathways.

Testing the superhydrophobic surfaces’ capability to create spheroid-like structures was conducted with the same initial cell densities as those examined in agarose-derived hydrogels (200 and 2000 cell/μL) over a 48 hour incubation period. The surfaces are very transparent, enabling direct observation of cell aggregates with optical microscopy. The results demonstrated that the characteristics of the formed spheroids were highly dependent on the cell concentration (Figure 2a). Lower concentration holds to the formation of fewer but denser aggregates, while at higher concentrations, spheroid formation appears to be almost inhibited.

Roundness plays a crucial role in spheroid analysis. Spherical clusters show in-creased stability in terms of morphological changes during growth, as well as in proliferative and necrotic areas [39]. This study assessed the geometric explanation of spheroid development by examining the circularity of the cross-sectional area, without assuming spherical symmetry in aggregate formation. Circularity is determined by dividing the shortest axis (d1) by the longest axis (d2) of a spheroid. Circularity values approaching 1.0 are suitable for a perfect circle, while values significantly deviating from 1.0 indicate elongated ellipsoids. As shown in Figure 2b, the hydrogel agarose-induced spheroids showing circularity values ranged between 0.85 and 0.86. The size of the resulting structures was always higher than 100 μm. No significant differences for both parameters were found as a function of initial cell density.

The circularity of cell aggregates on the superhydrophobic surfaces (SHS), as a measure of the effectiveness of 3D spheroid formation, was similar for the two assayed cell densities with values of 0.82 and 0.88 for initial cell densities of 200 and 2000 cel/μL, respectively, with significant differences (*p* < 0.01) between cell density values. As a general trend, and similar to that observed in agarose, the increase in cell density favored the formation of larger 3D aggregates. The size values of the generated aggregates (expressed in one dimension when considering their high circularity) were 100% for aggregates in the range of 50–100 μm, and 100% of the aggregates were >100 μm for initial cell densities of 200 and 2000 cel/μL, respectively. Significant differences (*p* < 0.05) between cell density values were found.

When the substrate effect on 3D spheroid formation was compared, significant differences (*p* < 0.05) were found between the circularity values for the aggregates generated at the highest cell density (2000 cel/μL). In the case of size distribution, significant differences were found in all cases (*p* < 0.05).

### 2.3. 2D Growth as a Measure of Cell Viability

**Figure 3:**
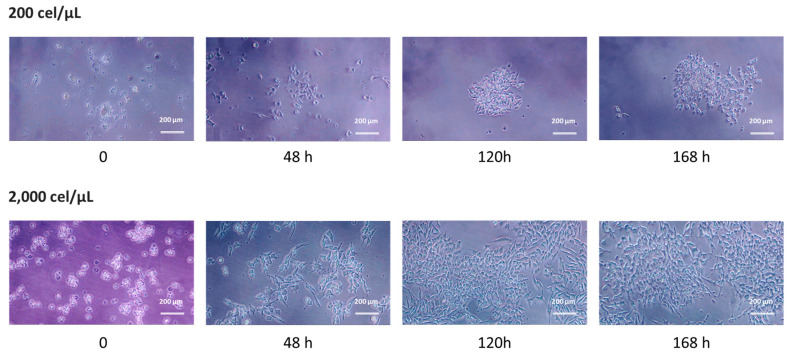
Phase contrast images of the temporal evolution) of cells derived from SHS-induced spheroids. Representative morphology of migrated cells at 168 h after recovery as a function of the initial cell density (200 or 2000 cel/μL). The scale bar represents 200 μm.

After assessing how superhydrophobicity affects the creation of 3D spheroids, it was crucial to assess the viability of the resulting cells. The culture medium had a high contact angle on these surfaces, indicating that it would be easy to remove the medium containing the 3D spheroids from surfaces by gentle handling. The migration capacity from the cell aggregate, adhesion, and growth under standard culture conditions was evaluated. Figure 3 shows representative images of this study based on the initial cell density and incubation time after recovery. The results showed how upon recovery, it was possible to visualize compacted aggregates depending on the initial cell density. By increasing the time (48 h), it could be observed how individual cells can be observed. Interestingly, these individual cells showed elongated shapes, especially in the case of migrated cells from SHS formed at the highest cell density. By increasing time, cells homogeneously distributed across the surface. The time required to form a monolayer depends on initial cell density on SHS-derived 3D aggregates.

### 2.4. Morphological Characterization of Migrated Cells from SHS

In this work, 3D profilometry was used to evaluate, qualitatively and quantitatively with nanometric resolution, the evident changes in the morphology of the cells transferred from the SHS-derived spheroids compared to control cells, that is, cells grown only under standard 2D monolayer conditions. Previous research conducted in our lab showed that this method can be seen as an attractive instrument for monitoring alterations in cell morphology while testing possible drugs and materials [40]. Moreover, a significant benefit of this method is its employment without the use of any fluorescent proteins or dyes, and without the spatial constraints (cm^2^) of other methods [41,42]. Once the influence of superhydrophobicity on the formation of 3D spheroids was assessed, it was essential to evaluate the viability on the derived cells. The high contact angle of the culture medium on these surfaces suggested that the medium containing the 3D spheroids could be easily isolated from surfaces under mild handling conditions. The migration capacity from the cell aggregate, adhesion, and growth under 2D standard culture conditions was evaluated. Figure 3 shows representative images of this study based on the initial cell density and incubation time after recovery. The results showed how upon recovery, it was possible to visualize compacted aggregates depending on the initial cell density. By increasing the time (48 h), it could be observed how individual cells can be observed. Interestingly, these individual cells showed elongated shapes, especially in the case of migrated cells from SHS formed at the highest cell density. By increasing time, cells momentously distributed across the surface. The time required to form a monolayer depends on initial cell density on SHS-derived 3D aggregates.

**Figure 4:**
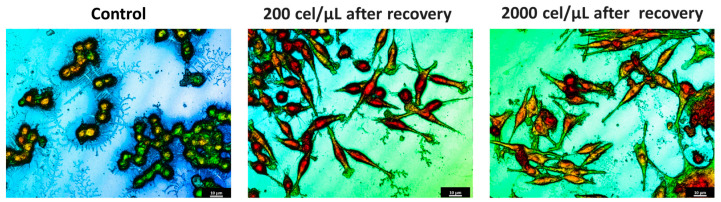
Three-dimensional optical profilometry images in confocal mode (100× magnification) for RAW264.7 spheroids formed on SHS surfaces depending on the initial cell density (200 or 2000 cel/μL) for 168 h after recovery and compared to control cells. The scale bar represents 10 μm.

At a qualitative level, this study showed how the migrated cells showed highly elongated morphologies than those derived from a conventional 2D monolayer culture (Figure 4). From a physicochemical point of view, these cellular entities would exhibit improved adhesion [43]. These results are in the light of recent results in our lab, in which 3T3 fibroblasts and HaCaT keratocytes derived from SHS-induced 3D spheroids revealed significant morphological changes [31].

It is well established that, depending on the shape, metabolism, and function, the macrophages are divided into three different subtypes. Native/inactivated M0 macrophages show round/slightly elongated morphology. Pro-inflammatory M1 macrophages, responsible to fight infections, are round. Oppositely, anti-inflammatory M2 macrophages that play a major role in tissue repair and wound healing are elongated [44]. A close look at the morphological characteristics of the migrated cells resembles macrophages of the M2 phenotype, which instead of the round-shaped morphology of control cells exhibited a very defined polarity with an expanded front and elongated tail. The physicochemical interactions between superhydrophobic surfaces and the cellular microenvironment play a crucial role in influencing macrophage polarization towards the M2 phenotype. These interactions could affect mechanotransduction pathways that are mediated by specific surface chemistries that impact cellular behavior. Among the different mechanisms, the surface roughness and surface chemistry of the proposed surperhydrophobic surfaces would mainly contribute to this polarization. The specific surface chemistry derived from the fluorinated compounds generate low surface energy materials, providing a highly hydrophobic environment that reduces protein adsorption and cell adhesion, thereby minimizing pro-inflammatory stimuli and promoting an anti-inflammatory phenotype [12,22].

Interestingly, the analysis of the profiles allowed us to evaluate numerical changes in the dimensions of the derived cells compared to the control cells (Table 1). Control cells exhibited sizes around 6.2–8.0 μm (axis length), providing a surface factor close to 1 as a consequence of the round-shaped structure. When migrated cells were considered, evident changes on the major axis length were determined. The values of major axis length ranged between 33 and 42 μm, or 52 and 90 μm, for systems at initial cell densities of 200 or 2000 cel/μL, respectively. To our knowledge, little evidence is present in the literature concerning the quantitative characterization of macrophages on the different morphological phenotypes. Nevertheless, these values are in the light of cell lengths on M2 phenotypes induced on bionanofilm substrates with different surface relief profiles [45] or macrophage polarization by magnetic interference [46]. Minor axis lengths were preserved (6.6–8 μm) with respect to control cells. The corresponding surface factor for the migrated cells showed values higher than 1, compatible with the loss of the round structure. Surface values ranged between 5 and 10 for migrated cells for systems at the initial cell densities of 200 or 2000 cel/μL, respectively. Significant differences between control cells (*p* < 0.05 and *p* < 0.001 for the initial cell densities of 200 or 2000 cel/μL, respectively) were found. Significant differences (*p* < 0.05) were also found between surface factor values of migrated cells at the two initial cell densities.

The analyses of the cell height demonstrated a slight reduction of the values in comparison with the control cells, compatible with the spread and elongation of the migration cells. Significant differences (*p* < 0.05) with respect control cells were found. No significant differences between height values at the two different cell densities were found. Concerning volume values, discrete changes were detected. No significant differences were found (neither between control cells nor between different cells densities). These results are in the light of the M2 phenotype. The activation toward an M1 phenotype generally increases cell volume due to their increased capacity for phagocytosis and cytokine production [18]. M2 macrophages do not focus as much on phagocytosis. However, their role in tissue repair and tissue remodeling requires cellular expansion and increased secretion capacity of growth factors and anti-inflammatory cytokines that also fit with a moderate increase in cell volume [44].

## 3. Discussion

Macrophages are essential components of the immune system. They are involved in preserving homeostasis, responding to infections, and supporting tissue repair. Their capability to adapt and react to diverse signals makes them crucial for maintaining health and fighting diseases such as cancer or chronic inflammatory diseases [47].

Elevated macrophage populations have been reported in malignant tumors, wound healing, bacterial infections, and other diseases. In the case of cancer, the crosstalk between different cells in the tumor microenvironment plays an important role in tumor growth and tumor-mediated immune suppression in vivo. Tumor-associated macrophages are abundant in most types of malignant tumors, which contribute to multiple cancer hallmark capabilities. To mimic these characteristics, spheroid models in coculture are widely used 3D tumor models, providing a 3D setting where immune cells can migrate toward and infiltrate tumor cell clusters [48,49]. Single culture of macrophages on 3D spheroids are less abundant in the literature. The recent studies of Burchett and coworkers have demonstrated the formation of RAW264.7 murine macrophage spheroids when embedded in agarose-based hydrogels [50]. The agarose concentration determined the properties of the spheroids. In 0.5% agarose, spheroids were much less regular in shape than those in 1% and 2%, resembling our results in 0.2% agarose-coating TCPS plates (Figure 2).

Superhydrophobic substrates have been found in the literature in hi-tech application to support spheroid growth. Nevertheless, one of the advantages shown in this work is the possibility to develop spheroid using any type of surface by a suitable functionalization with high reproducibility and as an easy-to-apply, low-cost solution. The selection of silica nanoparticles and fluorinated compounds might be considered cost-effective materials. The spray coating fabrication technique can also contribute to the scalability, adapted for large surfaces and multiple substrates. The effectiveness can be ensured by surface uniformity coating and quality control assessed by proper surface characterization techniques (CA, 3D profilometry). Studies on mechanical stability and environmental resistance [51] might ensure the final properties of the surfaces under various environmental conditions, such as exposure to UV light, humidity, and temperature variations. Using this technique, highly transparent glass surfaces with SH properties and homogeneous roughness have been coated (Figure 1). The highly hydrophobic contact angle observed at the culture liquid-coating interface is an effective condition for the 3D spheroid development during incubation of RAW 264.7 murine macrophages (48 h). The effect of two initial cell densities (200 and 2000 cel/μL) on the formation of the spheroids was investigated. The effect of substrate (agarose or SHS) demonstrated significant differences between the circularity values for the aggregates generated at the highest cell density (2000 cel/μL). In the case of size distribution, significant differences were found in all cases. These results suggested that SHS demonstrated improved features on the 3D aggregates as a function of the imposed compositions by the formation of denser, smaller aggregates compared to those formed on agarose hydrogel. The geometrical properties (circularity and size distribution) of the RAW264.7-prepared spheroids are comparable with our previous results on SHS-induced 3D aggregates [30,31]. In this case, the observed lower density and compactness could be associated with the involved cell line.

Although characterization techniques typically follow procedures developed for 2D cultures—where viability is determined by indirect, colorimetric assays [29]—the acridine orange/ethidium bromide (AO/EB) double-staining method has been used to describe SH-induced spheroids [30]. This method has shown a homogeneous distribution of viable cells, similar to the approach used by Burchett and coworkers [50]. In this work, following protocols previously developed in our lab [31], 3D aggregates were recovered and then grown under conventional 2D (monolayer) conditions to assess cell viability and migration (Figure 3). Over time, the cells remained viable and demonstrated migrating capabilities. Notably, the presence of elongated cells markedly differed from the round morphology typically observed in cells cultured under conventional 2D conditions.

Macrophage polarization is known to be accompanied by the change of cell shape [44]. Numerous studies have evaluated the macrophage phenotype after mechanical stimulation or by varying local stiffness [17,18,20]. M1 and M2 macrophages exhibited different degrees of elongation [52], and it was found that intended elongation of cells by the micropatterning of surface induced M2 polarization [17,44,52]. These studies highlight the role of cell shape in regulating macrophage function. In addition, recent studies in the literature demonstrated the impact of extracellular matrix (ECM) properties on macrophage polarization. Using engineered 3D collagen-based matrices mimicking the stiffness of dermis and connective tissues [19,24] or solid tumors [26], a strong impact on the polarization profile of macrophages to a M2-like phenotype have been described. The results in Figure 4 demonstrated the generation of spindle-like cells, relatively “short” cells with a pronounced central fusiform thickening of the body, for low initial cell density (200 cel/μL). However, by increasing initial cell density (2000 cel/μL), filiform cells with very long tails are dominant. Changes on the cell morphology upon spheroid formation are in light of our previous research [31]. Cells cultured on superhydrophobic surfaces produce ECM such as collagen, creating conditions suitable for cell proliferation and differentiation [53]. Our results suggested that cells on 3D spheroids ensure the cell–cell contact, resulting in M2-activated cells, especially for the migrated cells of SHS-derived 3D spheroids at the highest initial cell density.

Methods to induce macrophage polarization include the well-established cytokine treatment as well as biomaterial coating. More sophisticated techniques involve genetic engineering through CRISPR/Cas9 or other gene-editing techniques that can induce polarization. When factors such as cost, efficiency, and ease of implementation are compared, evidence demonstrates that superhydrophobic surfaces can be considered a promising tool for macrophage polarization, especially when long-term stability and sustained effects are desired. Although their implementation could require careful consideration of high initial costs and fabrication complexities compared to more straightforward methods like cytokine treatment, the chemical method can lead to some limitations when used for long-term applications. Genetic engineering would offer highly precise features but with complex and costly procedures. Biomaterial coatings are generally accessible but demonstrate variable efficiency. The approach explored in this work combines the advantages of both cost-effective materials (small quantities are required) and scalable preparation methods (spray coating method), which could improve the applicability of the derived surfaces compared with other induced-macrophage polarization methods.

Identifying macrophage phenotypes based on cytokine release is indeed a common method, but it can be both costly and time-consuming. To address these challenges, researchers have been exploring and developing alternative tools and techniques for macrophage phenotype identification. Biological analytical techniques have recently introduced Raman spectroscopy for its sensitivity beyond its non-destructive and non-invasive approach, with easier sample preparation [54]. In a similar way, 3D profilometry has been used here for characterizing migrated cell morphology at the nano-microscale through 3D profilometry (Figure 4, Table 1). Twofold are the innovative sides: Firstly, to the best of our knowledge, no evidence is reported about morphological characterization of cells forming [29] or derived [31] from 3D aggregates. Secondly, the non-invasive peculiarity of 3D profilometry has been demonstrated by the authors to be suitable for life sciences [55], showing that such a surface can be compared to common biochemical approaches for discriminating cells under control conditions from those with precursors of proliferation and cell death. A precise description requires both quantitative and qualitative examination of the derived formations. The assessment using 3D profilometry at the nano-microscale revealed the M2 phenotype of the cells with an elongated shape and increased surface factor, both qualitatively and quantitatively.

## 4. Materials and Methods

### 4.1. Materials

To create the superhydrophobic surfaces, fumed silica nanoparticles with dimethyl silylate group and primary particle size from 5 to 30 nm (EVONIK HDK H15) bought from Degussa (Hannover, Germany) were dispersed in a commercial fluoropolymer blend, used as received, containing a fluorosilane polymer (0.1 wt.%) in a hydrofluoroether solvent, methoxy-nonafluorobutane. SeaKem^®^ LE Agarose with gel strength (1%) ≥1200 g/cm^2^ was purchased from Lonza (Verviers, Belgium).

Dulbecco’s modified Eagle’s medium (DMEM) with high glucose content (4.5 g/L glucose), L-glutamine solution (200 mM), fetal bovine serum (FBS), penicillin–streptomycin solution (10,000 U/mL penicillin and 10 mg/mL streptomycin), trypsin–EDTA solution (170,000 U/L trypsin and 0.2 g/L EDTA), and phosphate-buffered saline (PBS) were purchased from Lonza. The 75 cm^2^ flasks and the culture dishes were obtained from TPP (Trasadingen, Switzerland).

### 4.2. Methods

#### 4.2.1. Surface Preparation and Characterization

For this study, the SH coating was applied directly on glass substate by spraying the dispersion of silica nano particles/commercial fluoropolymer blend in the concentration of 2.0 g/L. The coating was prepared by spraying several layers of dispersion, resulting in a material quantity ranging from 0.02 to 0.08 mg/cm^2^. The resultant SHS were examined to assess their high hydrophobicity and cleaned with deionized water to get rid of any physical adsorbed contaminants that might have an impact on cell viability. Surface wettability was studied through contact angle (CA) measurements at room temperature using an ASTRA view drop-shape tensiometer (developed at CNR-ICMATE [56] using MilliQ high-purity-grade water produced by an ion-exchange microfiltration system (Milli-Pore, Burlington, MA, USA). After gently placing 5 μL drops onto the surface, the CA was monitored until spreading equilibrium was reached. Additionally, the surface roughness (Sa) and structure of SH samples were assessed using 3D confocal and interferometric profilometry (Sensofar S-NEOX, Terrassa, Spain). The 3D profilometry, because of its quick and non-destructive use and characterization in compliance with ISO 25178, was selected to enable a large, scanned surface.

#### 4.2.2. Cell Cultures

Monocyte/macrophage-like cells (RAW 264.7 cell line) were provided by Dr. M Cristina Riera from the Department of Biology, Healthcare and the Environment at the Faculty of Pharmacy and Food Science (UB). Cells were grown in high-glucose DMEM medium supplemented with 10% (*v*/*v*) FBS, 1% (*v*/*v*) L-glutamine, and 1% (*v*/*v*) antibiotic mixture at 37 °C and 5% CO_2_. Cells were cultured in 75 cm^2^ culture flasks and routinely split when cells were approximately 80% confluent.

#### 4.2.3. Conventional 2D Culture

RAW 264.7 cells were seeded at 2 × 10^5^ cells/mL into 24-well cell culture plates for 48 h under 5% CO_2_ at 37 °C. Optical microscopy through a Nikon inverted microscope equipped with a video camera (Moticam 1080 HDMI&USB, Moticam 1080 HDMI and USB, Motic Europe, Barcelona, Spain) was used to monitor the cell morphology and growth. The image processor (Motic Images 3.0 software, Moticam 1080 HDMI and USB (Motic Europe, Barcelona, Spain) was used to analyze the obtained images.

#### 4.2.4. Cell Culture in Agarose-Induced 3D Culture

We coated 24-well cell culture plates with warm agarose solution (0.2 % (*w*/*v* in PBS) and left them to cool before they were used. Cells were seeded at two different cell densities (2 × 10^5^ cells/ mL and 2 × 10^6^ cells/mL). Cells were incubated for 48 h under standard conditions. The assessment of 3D aggregate formation was studied following the same procedure described in Section 4.2.3.

#### 4.2.5. Cell Culture in Superhydrophobic Substrates

Cells were seeded by confining the required volume into delimited areas of the coated samples fixed by a Viton, fluorinated elastomers O-ring to avoid the sample floating in the Petri dish.

Cells were seeded at two different cell densities (2 × 10^5^ cells/ mL and 2 × 10^6^ cells/mL). Cells were incubated for 48 h under standard conditions. The assessment of 3D spheroid formation was studied following the same procedure described in Section 4.2.3.

#### 4.2.6. Spheroid Recovery and Growth under 2D Conditions

To recover spheroids from SHS, the medium with the spheroids was gently removed three times before aspirating for delivery. The gathered spheres were evenly distributed in 24-well dishes with full DMEM solution at regular temperature and CO_2_ levels. Cell migration and proliferation were observed over time in standard monolayer conditions.

#### 4.2.7. Profilometry Studies

After elimination of the spent medium, cells were fixed with 4% (*v*/*v*) paraformaldehyde for 15 min. Fixed cells in sterile PBS and low temperature (approximately 5 °C) were maintained until the point to be scanned.

Confocal mode was used to analyze the morphology of the cells. Cells on selected areas were chosen, and the corresponding profiles analyzed with the SensoSCAN software 5.3 (Sensofar, Terrassa, Spain). Morphological parameters (surface factor, height, and volume) were compared with those derived from control cells.

#### 4.2.8. Statistical Analyses

IBM SPSS Statistics 29 software (IBM, Armonk, NY, USA) was used for statistical analyses. Differences between datasets were considered by one-way analysis of variance (ANOVA) following the Scheffé post hoc tests for multiple comparisons. Differences were considered statistically significant at *p* < 0.001 (unless otherwise mentioned). Results are reported as means ± standard deviation. Significant differences are highlighted with an asterisk or other symbols.

## 5. Conclusions

In this work, superhydrophobicity was used to prepare 3D spheroids from RAW 264.7 murine macrophages. This study demonstrates that while the geometrical properties, such as circularity and size distribution, of the RAW264.7 spheroids are consistent with previous findings on SHS-induced 3D aggregates, the observed lower density and compactness can be attributed to the specific cell line used. The features of the superhydrophobic surfaces must be tailored to improve the performance of the generated 3D spheroids. Nevertheless, the control of cell morphology through surface features presents a promising approach for the targeted regulation of cell differentiation and proliferation processes. By modulating surface properties, it is possible to influence macrophage phenotypes in a desired manner. The SH coating developed in this study underscores the importance of regulating macrophage polarization to achieve favorable outcomes. Our findings suggest that spheroid formation on highly repellent substrates induces the activation of M2-type RAW 264.7 cells. By promoting M2 polarization, the proposed surfaces might have significant implications for the translational potential for modulating immune responses and enhancing tissue regeneration from a cost-effective method with long-term effects.

Modulating the immune response to biomaterials by altering macrophage polarization has been shown to be an effective strategy for promoting tissue repair.

Potential off-target effects of biomaterials include the activation of unintended immune responses. By decreasing cell adhesion in these superhydrophobic surfaces, the release of proinflammatory cytokines might be avoided, further failing inflammation and chronic inflammatory states. The adsorption of proteins could activate the adaptative immune response through the activation of T cells or the production of antibodies against these proteins, potentially causing hypersensitivity reactions or allergic responses. The used fluorinated compound on these coatings provides a highly hydrophobic environment that reduces protein adsorption and cell adhesion, thereby minimizing pro-inflammatory stimuli and promoting an anti-inflammatory phenotype. Moreover, M2 macrophages secrete cytokines and growth factors that reduce fibroblast-mediated fibrosis and promote a regenerative response, leading to improved extracellular matrix (ECM) remodeling and tissue repair.

Due to the complexity of the immune system, a tissue regeneration model must benefit from the dynamic interplay between macrophages and other cell types, such as fibroblasts and endothelial cells. Ongoing research is focused on spheroids in co-culture conditions to study the interactions between different cell types within the aggregates. This approach can provide a more accurate and effective model for studying tissue regeneration and developing regenerative therapies.

## Figures and Tables

**Table 1:** Morphological phenotype characterization using 3D profilometry. Surface factor (major axis length/minor axis length ratio), height, and volume obtained from the corresponding cell profiles from RAW264.7 spheroids formed on SHS surfaces at two initial cell densities (200 or 2000 cel/μL) for 168 h after recovery and compared to the control cells. Results are reported as the average of ten independent cells ± standard deviation.

Profile	Surface Factor	Height (μm)	Volume (μm^3^)
**Control cells** 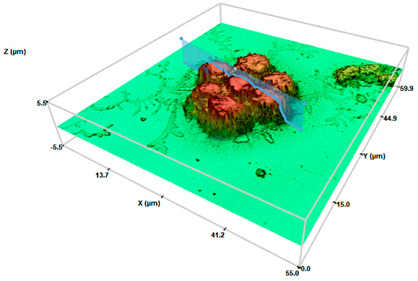	1.063 ± 0.031	4.50 ± 0.21	184 ± 0.201
**SHS 200 cel/μL** 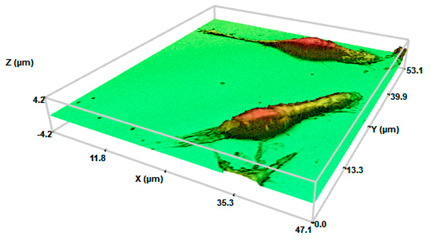	5.007 ± 0.193 *	3.2 ± 0.30 *	205 ± 0.471
**SHS 2000 cel/μL** 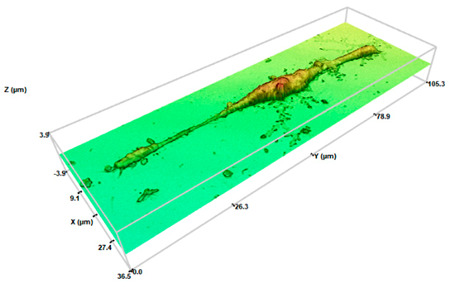	10.02 ± 2.21 **^●^	2.15 ± 0.65 **	223 ± 0.163

* *p* < 0.05 and ** *p* < 0.001 indicate significant differences between control cells. ^●^ *p* < 0.05 indicates significant differences between initial cell densities.

## Data Availability

Data are contained within the article.
